# A case report of acute carcinoid heart failure during lutetium-177 dotatate–triapine treatment for well-differentiated neuroendocrine tumors

**DOI:** 10.3389/fonc.2026.1801126

**Published:** 2026-05-08

**Authors:** Aman Chauhan, Elise C Kohn, S Percy Ivy, Jill M Kolesar, Rakhi Modak, Susanne Arnold, Lowell Anthony, William E. Carson, Nathan R Shelman, Charles A Kunos

**Affiliations:** 1 Division of Hematology/Oncology, Department of Medicine, University of San Francisco, San Francisco, CA, United States; 2 Cancer Therapy Evaluation Program, National Cancer Institute, Rockville, MD, United States; 3 College of Pharmacy, University of Kentucky, Lexington, KY, United States; 4 Department of Medical Oncology, University of Miami, Miami, FL, United States; 5 Division of Medical Oncology, Department of Internal Medicine, University of Kentucky, Lexington, KY, United States; 6 Translational Therapeutics, The Ohio State University, Columbus, OH, United States; 7 Department of Pathology and Lab Medicine, University of Kentucky, Lexington, KY, United States; 8 Department of Radiation Oncology, University of Miami, Miami, FL, United States

**Keywords:** [177Lu]lutetium-177 dotatate, carcinoid heart failure, neuroendocrine tumor, radiopharmaceutical, triapine

## Abstract

**Introduction:**

Well-differentiated neuroendocrine tumors (NETs) have a high indolent local progression rate when resistance develops to biologic or cytotoxic chemotherapy. Radiopharmaceuticals like single-agent [^177^Lu]lutetium-177 dotatate confer a 17% objective NET partial response rate with prolongation of progression-free survival.

**Case presentation:**

The patient, a 69-year-old man with gastroenteropancreatic NET with liver metastases and known chronic systolic heart failure (HF), had received octreotide then everolimus therapy prior to enrollment. At entry, his disease status was complicated by bilateral edema to his thighs worsening over a 4-week period and an echocardiogram showing right heart hypertrophic cardiomyopathy with an ejection fraction of 40%. [^68^Ga]Gallium-68 dotatate positron emission tomography identified tumors in the lung, liver, spleen, and axial skeleton. The phase I clinical trial involved intravenous [^177^Lu]lutetium-177 dotatate (day 1) given with concurrent once daily oral triapine (days 1–14) given as a radiosensitizer that repeated every 8 weeks for four cycles (NCT04234568); [^177^Lu]lutetium-177 dotatate requires concomitant treatment with 1 L of an isotonic amino acid salt solution, usually administered over a 4-h period around the radiopharmaceutical. On cycle 1 day 8, the patient was found unresponsive at home and efforts to resuscitate him were unsuccessful. Autopsy on cycle 1 day 9 revealed acute HF in the setting of HF complicated by carcinoid heart disease, measurable radionuclide organ uptake, and widely disseminated NET with 50% tumor lysis.

**Conclusion:**

This case reports the rare complication of an acute exacerbation of carcinoid-associated HF by the [^177^Lu]lutetium-177 dotatate–triapine combination. The cause of the acute cardiac decompensation could be related to the marked tumor response to treatment and/or contributions of the renoprotective amino acid infusion.

## Introduction

Metastatic neuroendocrine tumors (NETs) are uncommon cancers that arise from neoplastic cells of the normal diffuse neuroendocrine system coming predominantly (90%) from the gastroenteropancreatic (GEP) system (1). Metastatic NETs must be invasive and disseminated to the liver to give rise to the sequelae of carcinoid syndrome, which is often characterized by facial flushing, intractable diarrhea, and bronchoconstriction (2). Incident cases occur in as high as 10 per 100,000 persons (1); 50% of those with invasive metastases of the liver develop carcinoid syndrome (2). Of those who develop carcinoid syndrome and have invasive metastases of the liver, nearly 50% develop anatomic abnormalities of the right side of the heart (3), developing as a consequence of secreted vasoactive effectors like serotonin, histamine, tachykinins, or prostaglandins discharged into the circulation by the NET cells. This allows paracrine action on the heart and does not require direct NET cell invasion (3). Resultant potential heart abnormalities include fibrous tissue plaques of the tricuspid valve, pulmonary valve, cardiac chambers, venae cavae, pulmonary arteriae, and coronary sinus, all inducing distortion of the right-sided heart valves producing tricuspid regurgitation or both (2). One study found that the mean survival of those with carcinoid heart disease (1.6 years) was substantially shorter than those without such heart disease [4.6 years (3),]. The foremost prognostic factors in patients with carcinoid heart failure (HF) are right atrial or ventricular hypertrophy (88%) as well as right ventricular septal wall motion abnormalities (45%) (3).

Surgery is the foundation of treatment for resectable NET disease (4). Therapy directed to the liver may have palliative benefit and can attenuate downstream hormone-related effects (5–7). Somatostatin analogs relieve sequelae resulting from hormone hypersecretion in functional NETs and may delay disease progression in some patients (5). Chemotherapy disrupting angiogenesis, like sunitinib, or cell cycle progression, like everolimus, have been shown to prolong progression-free survival in patients with lung/pancreatic/thymic NETs, but are more limited therapeutic options in patients with NET developing carcinoid syndrome (6–8). [^177^Lu]lutetium-177 dotatate is the only radiopharmaceutical approved for second-line treatment of advanced-stage NETs (9–11), yielding small response rates (17%), long progression-free survival intervals, and clinically notable impact on overall survival (12).

The lack of overall survival or cure has led current research activity to focus on approaches to enhance [^177^Lu]lutetium-177 dotatate activity using novel potential radiosensitizing combinations. This case report describes an unexpected rapid clinical response and clinical decompensation and death in a patient receiving an investigational [^177^Lu]lutetium-177 dotatate–triapine combination.

## Case presentation

A 68-year-old man presented in January 2020 with 2-day duration of dyspnea and abdominopelvic imaging showing liver tumors of unknown etiology. Cardiac magnetic resonance imaging showed no infiltrative tumor or valve distortion, although it did reveal right heart hypertrophic cardiomyopathy, global hypokinesis, and a calculated ejection fraction of approximately 26%. Liver tumor biopsy demonstrated a well-differentiated grade 2 (Ki-67: 5%) NET with strongly positive immunohistochemical staining for synaptophysin and CD56. Baseline circulating chromogranin A was 13,160 ng mL^−1^. Octreotide scan in February 2020 showed only heterogeneous activity in the liver, confirming stage IV, cT1, cN0, cM1c well-differentiated (grade 2) presumed GEP NET.

First-line treatment involved subcutaneous octreotide depot injection (20 mg) resulting in a circulating chromogranin A reduction to 837 ng mL^−1^ (−94%) between February and April 2020. Chronic systolic HF improved, with an ejection fraction of 30% by echocardiogram in August 2020. Second-line every-other-day oral everolimus (10 mg) was introduced in November 2020 after three consecutive increases of circulating chromogranin A (peak 14,960 ng mL^−1^). Disease progression in the liver, pelvic lymph nodes, and axial skeleton coupled with a circulating chromogranin A of 62,900 ng mL^−1^ in February 2021 prompted change of treatment.

The now 69-year-old patient was screened for the investigational [^177^Lu]lutetium-177 dotatate–triapine combination in March 2021, at which time he had persistent watery stools and new lower-extremity swelling progressively worsening over a 4-week period. There were no constitutional symptoms such as fevers, chills, nausea or emesis, abdominal pain, myalgias, anorexia, or weight loss. His documented HF had improved by March 2021, with an ejection fraction of 40% with medication but there was worse right-sided disease with moderate tricuspid regurgitation and global hypokinesis but without morphological changes. He also had persistent anemia (hemoglobin 11.5 g/dL) and hypertension. His outpatient medications included furosemide (40 mg) daily, carvedilol (6.25 mg) one-half tablet twice daily, sacubitril/valsartan (24 mg/26 mg) twice daily, and aspirin (81 mg) daily. He was adopted; there was no known family history of neuroendocrine neoplasms.

**Figure 1:**
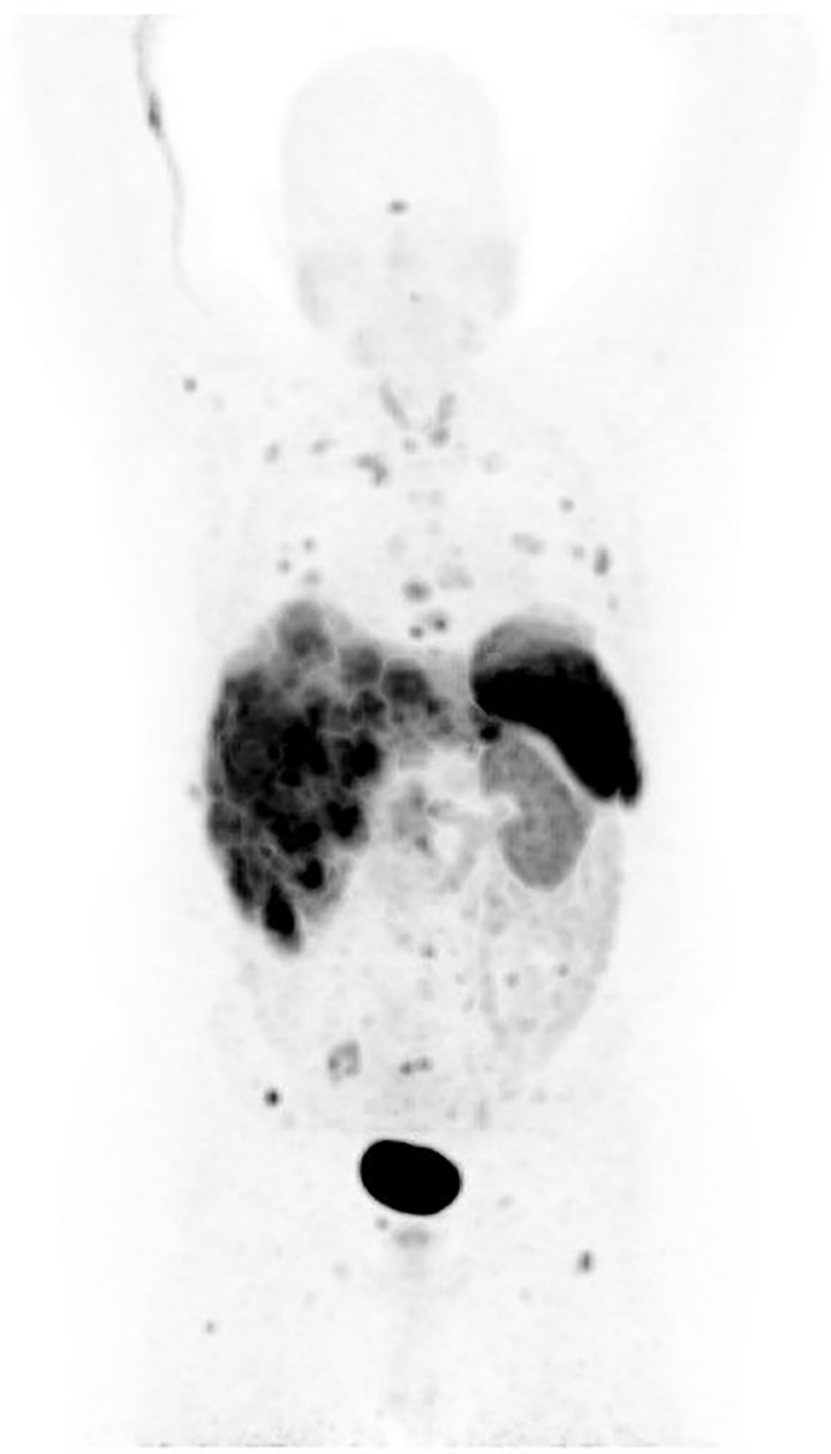
Nuclear medicine maximum intensity projection of patient disease burden at trial enrollment.

On baseline examination, he had bilateral 2+ pitting pedal edema extending to his mid-thighs but was able to conduct his activities of daily living without restriction (New York Heart Association class II). The remainder of the physical examination was unremarkable. He had an Eastern Cooperative Oncology Group performance status of 1. His chromogranin A concentration was 40,840 ng mL^−1^, and his serum serotonin level was 1,480 ng mL^−1^. Contrasted computed tomography images of the chest, abdomen, and pelvis in April 2021 were significant for diffuse heterogeneous metastatic liver involvement with no other discernable visceral disease. [^68^Ga]Gallium-68 dotatate positron emission tomography that same month identified tumors in the liver, spleen, lung, and axial skeleton (**Figure 1**).

He was eligible for and provided informed consent for the National Cancer Institute (NCI) Experimental Therapeutics Clinical Trial Network (ETCTN) protocol #10388 in May 2021. The trial was testing the addition of radiosensitizing oral 3-aminopyridine-2-carboxaldehyde-thiosemicarbazone (3AP, triapine) to the standard-of-care [^177^Lu]lutetium-177 dotatate for NETs (NCT04234568). His intended trial treatment was four cycles of [^177^Lu]lutetium-177 dotatate (200 mCi by vein day 1) with triapine (200 mg by mouth days 1–14) repeated every 8 weeks starting in May 2021 (dose level 3). He continued octreotide (30 mg intramuscular depot) monthly on trial per protocol for control of carcinoid syndrome.

**Figure 2:**
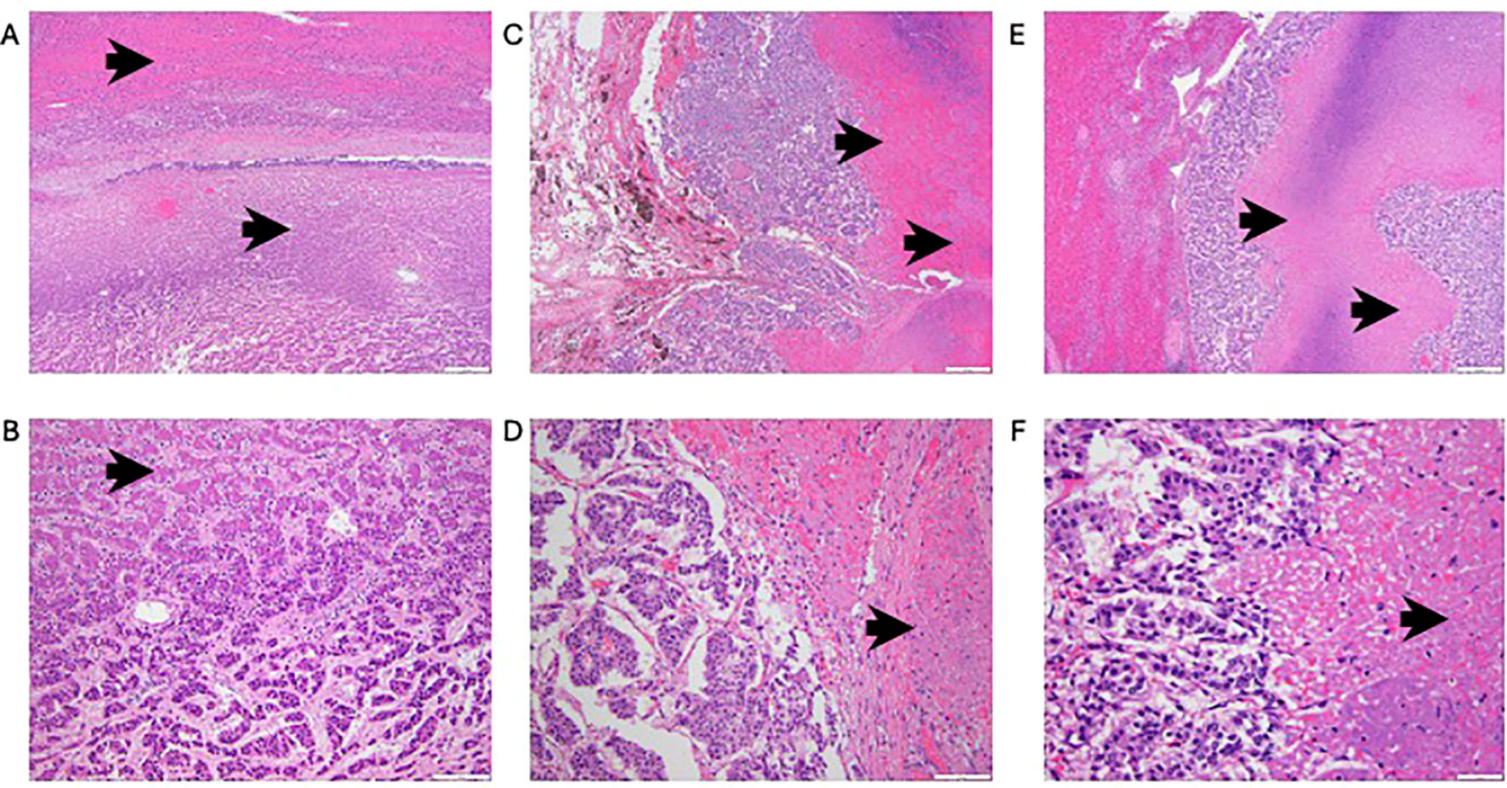
Photomicrographs of normal and necrotic neuroendocrine tumor from **(A, B)** liver, **(C, D)** lung, and **(E, F)** spleen. Black arrows indicate tumor necrosis. Images were acquired at ×40 (above) and ×200 magnification (below). Scale bars are as indicated.

Eight days after initiation of [^177^Lu]lutetium-177 dotatate–triapine therapy (cycle 1 day 8), he expired unexpectedly despite resuscitative effort by his wife. Autopsy done the following day identified the final diagnoses of acute HF in the setting of chronic HF, complicated by carcinoid heart disease, secondary to widely disseminated NET with 50% tumor lysis (**Figure 2**). The heart had hypertrophic cardiomyopathy, tricuspid valve fibrous plaques, and evidence of antecedent anterior and posterior wall ischemic infarcts. The lungs had pulmonary edema, a single right middle lode metastasis associated with alveolar hemorrhage, and no evidence of thromboembolic event. The liver was congested with 50% parenchymal nodular metastases measuring up to 7.5 cm in diameter. Splenomegaly was due to congestion and metastases measuring up to 3 cm in diameter. Acute renal tubular injury with mild patchy glomerulosclerosis was attributed to [^177^Lu]lutetium-177 dotatate renal elimination. The central nervous system was unremarkable. Multi-platform genomic methods (11) detected mutated *MEN1* expression, leading to impaired histone methyltransferase activity and microsatellite instability, reflecting a defective DNA mismatch repair pathway. **Table 1** lists NET and organ retention of [^177^Lu]lutetium-177 dotatate 8 days after injection and at autopsy.

**Table 1:** ^177^Lutetium dotatate organ and tumor tissue distribution in a human subject 8 days postinjection^1^.

Tissue	Tissue activity (nCi)	Tissue weight (g)	Tissue activity (nCi/g)	Tissue %ID per g (×10^-4^)	Organ weight (g)^2^	Organ activity (nCi)	Organ %ID
Blank	0.1261	0	0	0	0	0	0
Heart	5.6626	0.2256	24.54	0.12	570	13,988	0.007
Liver	36.8234	0.3054	120.16	0.60	1970	236,718	0.118
Liver tumor	934.1581	0.4176	2236.67	11.18	1970	4,406,233	2.203
Spleen	154.2297	0.4606	334.57	1.67	387	129,479	0.065
Right kidney	285.4229	0.3676	776.11	3.88	198	153,669	0.077

nCi, nanocurie; g, gram; %ID, percent of 200 millicurie injected dose × 100.

^1^ Cylindrical samples of relatively uniform geometry were collected from organs as well as liver tumor and counted for radioactivity in a well counter using a counting standard and a background standard. Tissue activity per gram is calculated by subtracting the background activity from tissue activity and dividing by the tissue weight. Organ activity is the product of tissue activity and organ weight.

^2^ Determined at autopsy within 24 h of death (cycle 1 day 9). Liver NET made up 50% of the organ at death; organ weight was halved in this calculation. Spleen organ weight is the sum of parenchyma and NET burden; a more sophisticated calculation cannot be done.

## Discussion

This case report describes acute toxicity during a clinical trial employing an investigational radiosensitizing strategy of a novel ribonucleotide reductase inhibitor, triapine, combined with standard-of-care [^177^Lu]lutetium-177 dotatate radiopharmaceutical administration for the management of metastatic NETs, which was associated with a presumed treatment-related acute exacerbation of carcinoid HF despite trial protocol-directed somatostatin analog therapy. The acute cardiac decompensation could have been caused by rapid tumor response to treatment with tumor lysis-associated release of vasoactive neuroendocrine factors, coupled with impact of the fluid load required for [^177^Lu]lutetium-177 dotatate renal protection. Persistent disease after cytotoxic and cytostatic therapies associates with a high local tumor recurrence rate [82% (12),] and, thus, motivates the advance of original radiopharmaceutical–radiosensitizing drug combinations in early-phase clinical trials. In the example of [^177^Lu]lutetium-177 dotatate–triapine, there was an overwhelming tumor response with lysis of NET cells, possibly resulting in sequential events of elevated blood potassium, dysrhythmia, and exacerbated systolic HF. While causation is difficult to determine, the timing and sequence of tumor lysis followed by a cardiac event contend that the observed adverse event associated to the investigational combination used to treat the patient’s NETs.

The management of patients with NET with suspected carcinoid heart disease remains multifaceted because both the metastatic disease and the cardiac status need to be addressed simultaneously (8, 13). Medical management of right HF in this clinical setting (i.e., prior to planned radiopharmaceutical therapy) usually involves loop diuretics plus aldosterone receptor antagonists. Diuretics alleviate edema and hepatic congestion resulting from the carcinoid heart disease, albeit with the diuretics elevating the hazard for intravascular depletion and for decrease in cardiac output. For [^177^Lu]lutetium-177 dotatate treatment, renoprotective amino acid infusion raises plasma volume; however, the excess plasma volume might necessitate additional doses of loop diuretics. Digoxin might be helpful for these patients demonstrating reduced right ventricular contractility (2). In those patients with severe symptomatic HF (i.e., fatigue, dyspnea, edema, and ascites) with 12 months or more of predicted post-operative NET-related survival, cardiac surgery, with the choice of valve prosthesis individualized, may be indicated (8). Single valve transcatheter replacements have been reported for high-risk patients with carcinoid heart disease (14). Selection of patients with NET with suspected carcinoid heart disease for peptide receptor radionuclide therapy alone or in combination requires careful clinical consideration.

Also, of interest, the [^177^Lu]lutetium-177 dotatate–triapine combination was experimental and studied on clinical trial with appropriate informed consent procured from the patient. A phase I trial continuously evaluates observed toxicity to determine the appropriateness of drug dose (de)escalation. This trial deviates from the usual decision-making process in that only a single subject (i.e., this patient) was entered at the highest dose level. Data from this patient and use of safety data from an external early-phase trial conducted in another disease type prompted de-escalation, which confuses any capability to analyze this trial’s originally planned radiopharmaceutical-drug dose-toxicity model. Moreover, the patient here had an unexpected exceptional response given that he had 50% tumor lysis after only 8 days of exposure to the [^177^Lu]lutetium-177 dotatate–triapine combination. Postmortem retention of [^177^Lu]lutetium-177 dotatate radioactivity confirmed localization of radiopharmaceutical in the intended NET targets like the liver but not the heart muscle.

## Conclusions

Given that metastatic progressive NETs associate with a poor prognosis in at least partly due to a high local recurrence rate, new therapeutic strategies studied in early-phase clinical trials remain attractive. This case report describes a relatively rare toxicity of acute carcinoid HF, definitely attributed to a [^177^Lu]lutetium-177 dotatate–triapine combination. In next trial iterations of the [^177^Lu]lutetium-177 dotatate–triapine combination, a consideration for an eligibility exclusion criterion with defined acceptable parameters for symptomatic carcinoid HF should be made. Further research of dual-agent radiopharmaceutical–radiosensitizing drug combinations in phase I trials should be operationalized and activated for patients with metastatic NETs.

## Data Availability

The raw data supporting the conclusions of this article will be made available by the authors, without undue reservation.

## References

[1] Chauhan A Kohn E Del Rivero J . Neuroendocrine tumors-less well known, often misunderstood, and rapidly growing in incidence. JAMA Oncol. (2020) 6:21–2. doi: 10.1001/jamaoncol.2019.4568. PMID: 31697337 PMC9382718

[2] Fox DJ Khattar RS . Carcinoid heart disease: presentation, diagnosis, and management. Heart. (2004) 90:1224–8. doi: 10.1136/hrt.2004.040329. PMID: 15367531 PMC1768473

[3] Pellikka PA Tajik AJ Khandheria BK Seward JB Callahan JA Pitot HC . Carcinoid heart disease. Clinical and echocardiographic spectrum in 74 patients. Circulation. (1993) 87:1188–96. doi: 10.1161/01.cir.87.4.1188. PMID: 7681733

[4] Doherty G . Surgical treatment of neuroendocrine tumors (including carcinoid). Curr Opin Endocrinol Diabetes Obes. (2013) 20:32–6. doi: 10.1097/MED.0b013e32835b7efa. PMID: 23183358

[5] Anthony L Freda PU . From somatostatin to octreotide LAR: evolution of a somatostatin analogue. Curr Med Res Opin. (2009) 25:2989–99. doi: 10.1185/03007990903328959. PMID: 19842996 PMC3678951

[6] Raymond E Dahan L Raoul JL Bang YJ Borbath I Lombard-Bohas C . Sunitinib malate for the treatment of pancreatic neuroendocrine tumors. N Engl J Med. (2011) 364:501–13. doi: 10.1056/NEJMoa1003825. PMID: 21306237

[7] Pavel ME Hainsworth JD Baudin E Peeters M Hörsch D Winkler RE . Everolimus plus octreotide long-acting repeatable for the treatment of advanced neuroendocrine tumours associated with carcinoid syndrome (RADIANT-2): a randomised, placebo-controlled, phase 3 study. Lancet. (2011) 378:2005–12. doi: 10.1016/s0140-6736(11)61742-x. PMID: 22119496

[8] Grozinsky-Glasberg S Davar J Hofland J Dobson R Prasad V Pascher A . European Neuroendocrine Tumor Society (ENETS) 2022 guidance paper for carcinoid syndrome and carcinoid heart disease. J Neuroendocrinol. (2022) 34:e13146. doi: 10.1111/jne.13146. PMID: 35613326 PMC9539661

[9] Strosberg J El-Haddad G Wolin E Hendifar A Yao J Chasen B . Phase 3 trial of (177)Lu-dotatate for midgut neuroendocrine tumors. N Engl J Med. (2017) 376:125–35. doi: 10.1056/NEJMoa1607427. PMID: 28076709 PMC5895095

[10] Brabander T van der Zwan WA Teunissen JJM Kam BLR Feelders RA de Herder WW . Long-term efficacy, survival, and safety of [(177)Lu-DOTA(0),Tyr(3)]octreotate in patients with gastroenteropancreatic and bronchial neuroendocrine tumors. Clin Cancer Res. (2017) 23:4617–24. doi: 10.1158/1078-0432.Ccr-16-2743. PMID: 28428192

[11] Das S Chauhan A Du L Thomas KE Jacob A SChad A . External validation of a clinical score for patients with neuroendocrine tumors under consideration for peptide receptor radionuclide therapy. JAMA Netw Open. (2022) 5:e2144170. doi: 10.1001/jamanetworkopen.2021.44170. PMID: 35044469 PMC8771294

[12] Strosberg JR Caplin ME Kunz PL Ruszniewski PB Bodei L Hendifar A . (177)Lu-dotatate plus long-acting octreotide versus high-dose long-acting octreotide in patients with midgut neuroendocrine tumours (NETTER-1): final overall survival and long-term safety results from an open-label, randomised, controlled, phase 3 trial. Lancet Oncol. (2021) 22:1752–63. doi: 10.1016/s1470-2045(21)00572-6. PMID: 34793718

[13] Hicks RJ Kwekkeboom DJ Krenning E Bodei L Grozinsky-Glasberg S Arnold R . ENETS consensus guidelines for the standards of care in neuroendocrine neoplasia: peptide receptor radionuclide therapy with radiolabeled somatostatin analogues. Neuroendocrinology. (2017) 105:295–309. doi: 10.1159/000475526. PMID: 28402980

[14] Barbier MA Gerard L Grinberg D François L Walter T Obadia JF . Transcatheter valve replacement in carcinoid heart disease: a potential change of paradigm. Interdiscip Cardiovasc Thorac Surg. (2026) 41. doi: 10.1093/icvts/ivag001. PMID: 41506885 PMC12836423

